# Analysis of the High-Performance Liquid Chromatography Fingerprints and Quantitative Analysis of Multicomponents by Single Marker of Products of Fermented *Cordyceps sinensis*

**DOI:** 10.1155/2018/5943914

**Published:** 2018-04-10

**Authors:** Li-hua Chen, Yao Wu, Yong-mei Guan, Chen Jin, Wei-feng Zhu, Ming Yang

**Affiliations:** ^1^Key Laboratory of Modern Preparation of TCM, Ministry of Education, Jiangxi University of Traditional Chinese Medicine, No. 18 Yun Wan Road, Nanchang 330004, China; ^2^Jiangxi Sinopharm Co. Ltd., No. 888 National Medicine Road, Nanchang 330004, China

## Abstract

Fermented *Cordyceps sinensis*, the succedaneum of *Cordyceps sinensis* which is extracted and separated from *Cordyceps sinensis* by artificial fermentation, is commonly used in eastern Asia in clinical treatments due to its health benefit. In this paper, a new strategy for differentiating and comprehensively evaluating the quality of products of fermented *Cordyceps sinensis* has been established, based on high-performance liquid chromatography (HPLC) fingerprint analysis combined with similar analysis (SA), hierarchical cluster analysis (HCA), and the quantitative analysis of multicomponents by single marker (QAMS). Ten common peaks were collected and analysed using SA, HCA, and QAMS. These methods indicated that 30 fermented *Cordyceps sinensis* samples could be categorized into two groups by HCA. Five peaks were identified as uracil, uridine, adenine, guanosine, and adenosine, and according to the results from the diode array detector, which can be used to confirm peak purity, the purities of these compounds were greater than 990. Adenosine was chosen as the internal reference substance. The relative correction factors (RCF) between adenosine and the other four nucleosides were calculated and investigated using the QAMS method. Meanwhile, the accuracy of the QAMS method was confirmed by comparing the results of that method with those of an external standard method with cosines of the angles between the groups. No significant difference between the two methods was observed. In conclusion, the method established herein was efficient, successful in identifying the products of fermented *Cordyceps sinensis*, and scientifically valid to be applicable in the systematic quality control of fermented *Cordyceps sinensis* products.

## 1. Introduction

In recent years, fermented *Cordyceps sinensis*, as a substitute for natural *Cordyceps sinensis*, has been attracting increasing attention from across the globe. Some of the major products prepared from fermented *Cordyceps sinensis* include Jinshuibao capsules, Jinshuibao tablets, and Bailing capsules. Modern pharmacological studies have demonstrated that this material has antioxidative, anticancer [1], antihyperglycaemic [2], and antifatigue [3] properties, and it has a similar chemical composition and pharmacological activities to *Cordyceps sinensis*. Moreover, modern pharmacological and clinical studies have also revealed that fermented *Cordyceps sinensis* can have a major impact on the treatment of chronic hepatitis and kidney failure. These curative effects could be attributed to their active chemical components, including nucleosides, polysaccharides, sterols, amino acids, flavonoids, and metal complexes [4–8]. The type and quantity of the bioactive ingredients in fermented *Cordyceps sinensis* vary with the strain and the condition of the material [9, 10]. Among these components, nucleosides support the regulation and modulation of various physiological processes in the body [11], and they have been recognized as the main bioactive components [12] in fermented *Cordyceps sinensis*. Their pharmacological activities include antitumour, antileukaemic [13], antiviral [14], and anti-HIV [15] activities. The major nucleosides in fermented *Cordyceps sinensis* are guanosine, uridine, adenosine, uracil, and adenine.

Currently, the products of fermented *Cordyceps sinensis* have no uniform quality standards, which have resulted in the flooding of the market with products of uneven quality. Only Jinshuibao capsules, Jinshuibao tablets, and Bailing capsules, which are prepared by fermenting with *Paecilomyces hepiali* chen and *Hirsutella sinensis* Liu, *GUO*, *Yu-et Zeng*, have been recorded in Chinese Pharmacopoeia and have relatively comprehensive quality standards. The quality of the three fermented *Cordyceps sinensis* products is higher overall and is easier to control since they are produced exclusively by one company. The quality standards for fermented *Cordyceps sinensis* products vary from one manufacture to another. For instance, in the Pharmacopoeia of the People's Republic of China (Edition 2015) [16], the quality standards for Jinshuibao capsules included the identification of five required nucleosides, amino acids, and mannitol as well as required contents of adenosine and ergosterol. The standards for Bailing capsules include the identification of two required nucleosides, amino acids, and ergosterol as well as required contents of mannitol, adenosine, and total amino acid. Thus, the development of a method to control the quality of these products more systematically and effectively is an urgent issue. Since the overall chemical composition is contained within the chromatographic fingerprint, this type of fingerprinting may provide the foundation for such method. Meanwhile, chromatographic fingerprinting has been regarded as a feasible and rational approach to the quality evaluation of crude drug materials in recent years [17]. In addition, methods based on this technique have been accepted by the WHO, the FDA, and the State Food and Drug Administration (SFDA) of China [18]. However, chemical fingerprinting is a type of qualitative analysis, and thus, it can only reflect the general characteristics of the contents of herbs and serve as an indicator of their quality, consistency, and stability. Thus, the cataloguing and evaluation of chromatographic fingerprints are of great importance to evaluating and distinguishing medicinal materials and related preparations.

Multicomponent quantitative analysis was developed for applications in comprehensive quality control. Unfortunately, the use of multicomponent quantitative analysis is limited by the high price, limited availability, and poor stability of some of the necessary standards. Thus, a QAMS method was carried out. A predominant advantage of this strategy is that the content of each target component can be determined independently since their concentrations can be calculated based on standard materials. In recent years, the QAMS method has been widely used to evaluate a large number of Chinese herbal medicines [19–21] and was reported in the Chinese Pharmacopoeia to be applicable to the determination of the contents of TCM herbs, including *Coptis chinensis*, *Salvia miltiorrhiza*, and *Ganoderma lucidum* [16].

However, the current method was not sufficient to fully assess the quality of products of fermented *Cordyceps sinensis*. In this work, a partial method involving analysis of HPLC fingerprint chromatograms by SA and HCA combined with QAMS was established for the first time. Adenosine was chosen as the internal reference substance, and the relative correction factors (RCF) between the internal marker and test specimen were used to calculate the other four components. These steps in combination with HPLC fingerprint analysis based on SA and HCA can be used to obtain more general information on the products of fermented *Cordyceps sinensis*. The rapid, practical, and effective combinatorial approach can be used to comprehensively assess the quality of products prepared from fermented *Cordyceps sinensis*.

## 2. Materials and Methods

### 2.1. Reagents and Chemicals

Four reference substances (guanosine, adenosine, uracil, and adenine) were purchased from the National Institutes for Food and Drug Control (China), and uridine was purchased from the Nanchang Beta Biotechnology Co. Ltd. 10 Jinshuibao capsules (strain: *Paecilomyces hepiali* chen, batch number: 1,60,40,185, 1,50,50,347, 1,60,50,229, 1,60,20,075, 1,60,30,133, 1,60,90,394, 1,51,10,701, 1,60,80,393, 1,60,10,105, and 1,60,70,345) and 10 Jinshuibao tablets (strain: *Paecilomyces hepiali* chen, batch number: 1,60,703, 1,60,601, 1,60,401, 1,60,701, 1,60,501, 1,60,702, 1,61,202, 1,61,203, 1,61,204, and 1,60,502) were purchased from Jiangxi Jimingkexin Jinshuibao Co. Ltd., and 10 Bailing capsules (strain: *Hirsutella sinensis* Liu, *GUO*, *Yu-et Zeng*, batch number: 16,04,172, 1,6,04,101, 16,04,137, 16,05,171, 16,03,200, 16,03,102, 16,05,172, 16,06,124, 16,06,203, and 16,06,106) were purchased from Hangzhou Huadong Pharmaceutical Co. Ltd. The purity of each of the 5 reference substance was more than 98%. HPLC grade methanol was purchased from TEDIA reagent company (USA).

### 2.2. Instruments and Chromatographic Conditions

**Figure 1:**
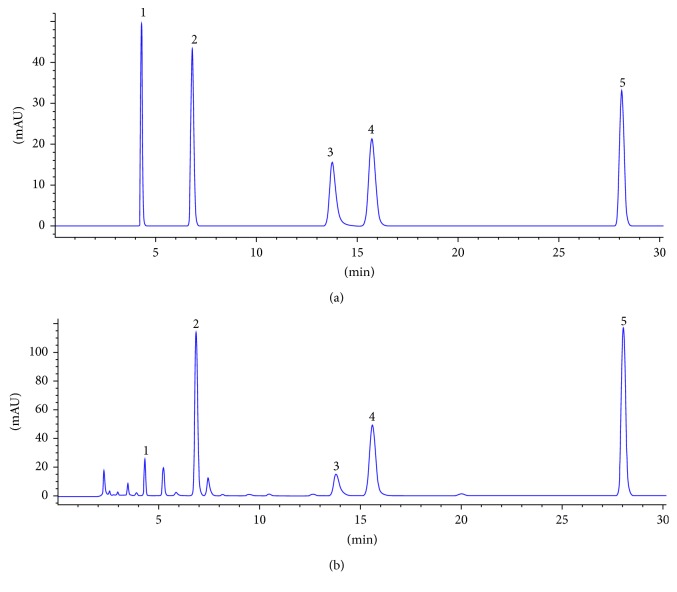
A chromatogram of the mixture of the standard compounds (a), and a chromatogram of fermented *Cordyceps sinensis* in Jinshuibao capsules (b). Peak 1: uracil; peak 2: uridine; peak 3: adenine; peak 4: guanosine; and peak 5: adenosine.

Chromatographic separations were performed on an Agilent 1260 HPLC coupled with a DAD, an Agilent 1100 HPLC coupled with a UV detector, and a Waters 2695 HPLC coupled with a PDA detector. The separations of the analytes were achieved on a reversed-phase C18 column (Agilent ZORBAX-RP, 250 × 4.6 mm, 5 *µ*m; Phenomenex OOG-4435-EO, 250 × 4.6 mm, 5 *µ*m). A sample volume of 10 *µ*L was injected by an autosampler, and the column temperature was maintained at 30°C. The mobile phase was composed of ultrapure water (A) and methanol (B) with a gradient elution system as follows: 0–15 min, 1% B; and 15–30 min, 1%–15% B. As show in Table 1, the flow rate was 1 mL/min, and the wavelength of the DAD was set at 260 nm. Under the chromatographic conditions described above, all five components could be baseline separated within 30 min (Figure 1).

### 2.3. Preparation of Standard Solutions

Five stock solutions were prepared by dissolving the requisite components in ultrapure water. A working solution that contains the five standard solutions was prepared prior to analysis. The concentrations of the components in the mixed solution were as follows: uracil 33.8 *µ*g/mL, uridine 93.6 *µ*g/mL, adenine 30.1 *µ*g/mL, guanosine 120.8 *µ*g/mL, and adenosine 93.6 *µ*g/mL.

### 2.4. Preparation of Sample Solution

A 1.00 g sample of the fermented *Cordyceps sinensis* was precisely weighed and immersed in 40 mL of ultrapure water in a 50 mL volumetric flask. The total mass of the flask and sample was determined, and the flask was placed in an ultrasonic bath for 30 min. The procedure was repeated twice. After sonication, additional ultrapure water was added to return the sample to its original weight. The mixture was separated by centrifugation, and the extracted solution was cooled to room temperature. Next, the diluted extract was filtrated through a 0.22 *µ*m membrane filter into an HPLC vial to prepare it for HPLC analysis.

### 2.5. Similar Analysis (SA)

The similarity evaluation method for the chromatographic fingerprints of TCM (Version 2004A) recommended by the SFDA of China was used to obtain standardized fingerprint chromatograms of three kinds of fermented *Cordyceps sinensis*. Acquiring the standardized fingerprint chromatograms involved the calibration and normalization of the retention times of all the common peaks of the three forms of fermented *Cordyceps sinensis*.

### 2.6. Hierarchical Cluster Analysis (HCA)

The similarity or dissimilarity of each sample was visually displayed with a dendrogram in HCA. In this paper, samples of different products prepared from fermented *Cordyceps sinensis* were compared using SPSS 21.0 software (IBM company, New York, America) based on the between-groups linkage method and the cosine of the angular distance.

### 2.7. Calculation of Relative Conversion Using the QAMS Method

In a certain linear range, the concentration of a component is proportional to the response of the detector. Adenosine was selected as the internal reference analyte because it is stable, readily available, inexpensive, and present in a high concentration in Jinshuibao capsules. The RCFs between adenosine and the other analytes were determined using (1). The contents of other five components can be calculated using (2) and (3).

(1) $$\begin{array}{c} F_{i/s}=\frac{f_{i}}{f_{s}}=\frac{C_{i}}{C_{s}}\times \frac{A_{s}}{A_{i}}, \end{array}$$

(2) $$\begin{array}{c} C_{i}=\frac{f_{i}}{f_{s}}\times \frac{A_{i}}{A_{s}}\times C_{s}, \end{array}$$

(3) $$\begin{array}{c} m_{i}=C_{i}\times V_{i}, \end{array}$$

where *A*_i_ is the peak area of the analyte, *A*_s_ is the peak area of the standard solution, *C*_i_ is the concentration of the analyte, *C*_s_ is the concentration of the standard, and *m*_i_ is the mass of the analyte in the capsule or tablet.

## 3. Results and Discussion

### 3.1. Method Validation

*Calibration Curves, Limits of Detection, and Limits of Quantity*. The linearity of the calibration curves were determined by plotting the results from six standard solutions at different concentrations. The standard curve and linear range were obtained as shown in Table 2. There was a good linear relationship over a wide concentration range. The LODs and the LOQs of the five analytes were determined at the noise-signal ratios of 3 : 1 and 10 : 1, within the ranges of 0.005–0.038 *µ*g/mL and 0.025–0.151 *µ*g/mL, respectively.

### 3.2. Precision Stability, Repeatability, and Recovery

The precision of the method was validated by their intraday and interday variability. The intraday precision was evaluated by injecting the same standard solution for six times. The interday variability was evaluated on 3 successive days using the same standard solution. The stability of the sample solutions was analysis at 0, 2, 4, 6, 8, 10, and 24 h. The sample solutions were found to be stable for 24 h (RSD ≤ 0.48%). To validate the repeatability of the method, five different sample solutions from the same sample were injected into the HPLC system, and the RSD values were found to be 0.23–1.36%. Recovery was evaluated by adding the same amount of an individual standard into a certain amount (0.2 g) of fermented *Cordyceps sinensis* sample that was found to have a concentration in the middle of the linear range (this procedure was repeated six times). In addition, the data from the DAD on the purities of the peaks indicate that the concentrations of the five nucleosides were not beyond the range of the threshold value, and the peak purity index of each compound was more than 990. This result confirmed that the purities of the chromatographic peaks were good, and the method is sufficiently accurate. All the results are showed in Table 3 and suggest that these methods are reliable and effective.

### 3.3. HPLC Fingerprint Analysis

**Figure 2:**
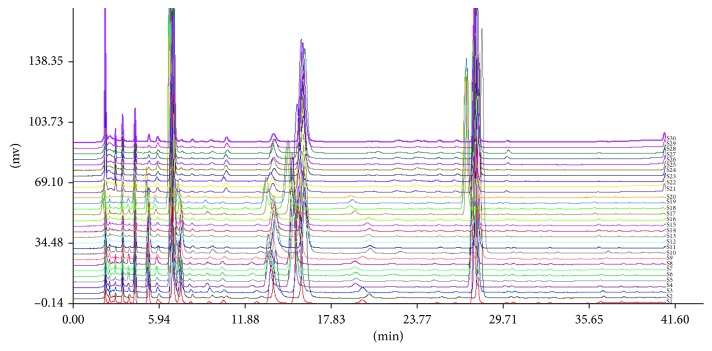
S1–S10: Jinshuibao capsule; S11–S20: Jinshuibao tablet; and S21–S30: Bailing capsule.

**Figure 3:**
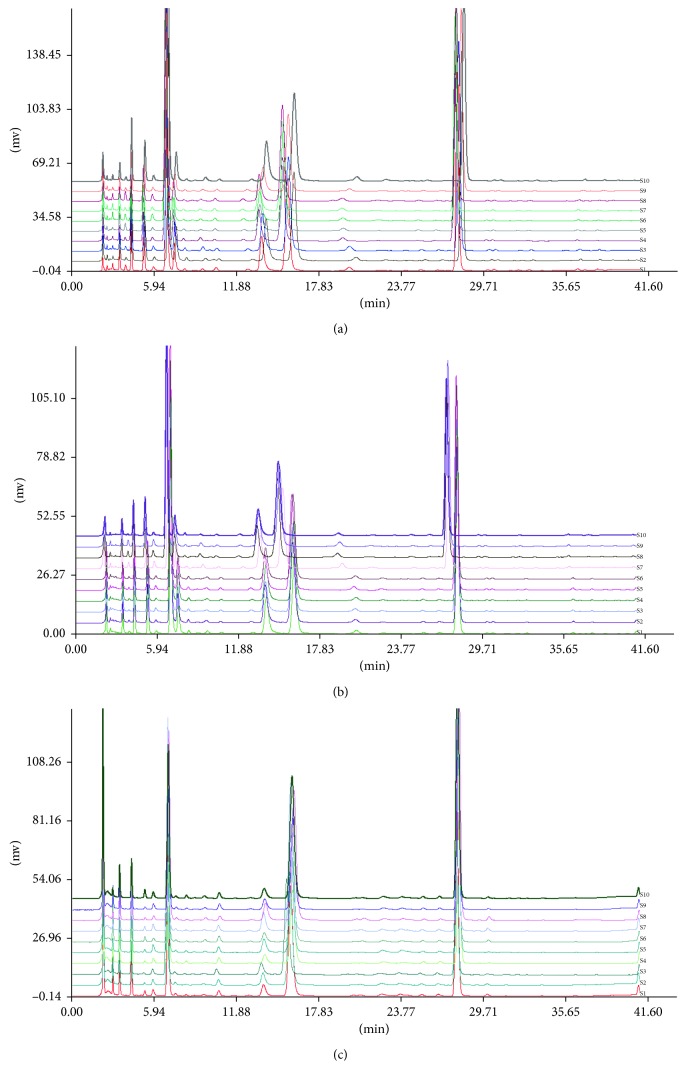
HPLC fingerprints for 30 fermented *Cordyceps sinensis* samples: 10 Jinshuibao capsule samples (a), 10 Jinshuibao tablet samples (b), and 10 Bailing capsule samples (c).

**Figure 4:**
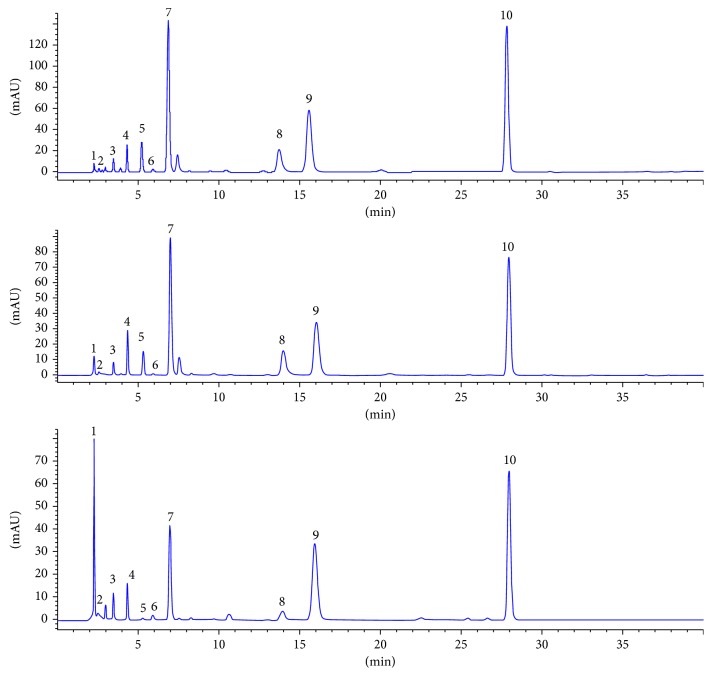
Common model fingerprints of Jinshuibao capsules (S1), Jinshuibao tablets (S2), and Bailing capsules (S3).

The fingerprint chromatograms of all three fermented *Cordyceps sinensis* products (Figure 2) and the fingerprint chromatograms of each sample (Figure 3) were established. The common patterns of each fermented *Cordyceps sinensis* were established according to the relative times and peak areas (RRTs and RPAs, resp.) of 10 characteristic peaks (Figure 4). Furthermore, peaks 4, 7, 8, 9, and 10 were identified as uracil, uridine, adenine, guanosine, and adenosine, respectively. Bailing capsules had the lowest contents of the compounds with retention times of 5.3 min, 13.5 min, and 20 min (almost no absorption), and Jinshuibao tablets had the lowest content of the compound with a retention time of 3.0 min (Figure 3). The similarity values of the Jinshuibao capsules, Jinshuibao tablets, and Bailing capsules were 0.988, 0.990, and 0.994, respectively. The correlation coefficients between each chromatogram of the fermented *Cordyceps sinensis* samples and the simulated mean chromatogram were 0.998, 0.996, 0.996, 0.997, 0.998, 0.999, 0.998, 0.998, 0.999, 0.989, 0.993, 0.993, 0.995, 0.993, 0.994, 0.997,0.981, 0.980, 0.981, 0.994, 0.978, 0.983, 0.984, 0.977, 0.975, 0.981, 0.985, 0.986, 0.979, and 0.974. These coefficients indicate that the samples from different companies are all of uniformity good quality since they are produced by the same method. In addition, the similarities of the samples fermented using the same strain and samples from the same company were fairly close.

### 3.4. Hierarchical Cluster Analysis (HCA)

**Figure 5:**
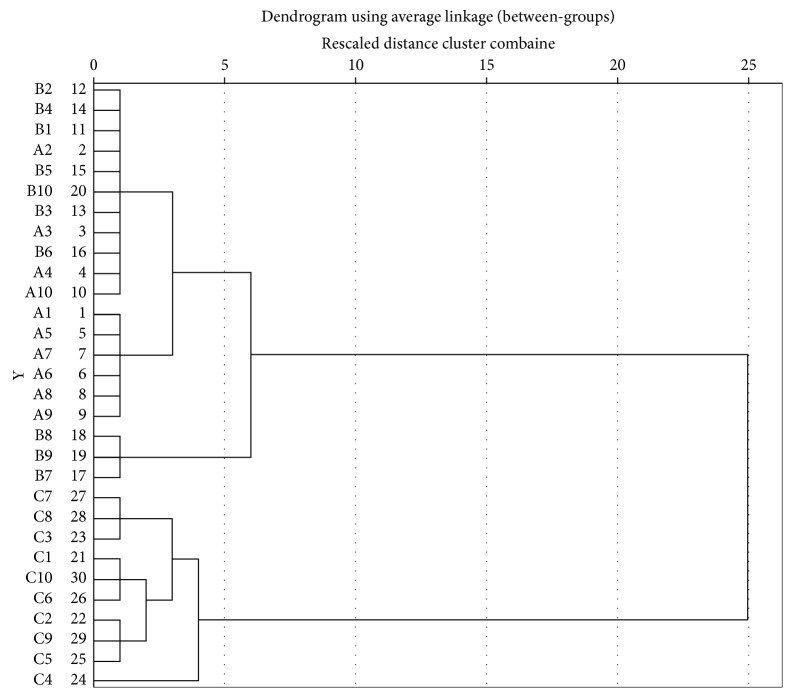
Clustering analysis graph of 30 fermented *Cordyceps sinensis* samples.

HCA on the 30 samples was carried out using SPSS 21.0 software based on the between-groups linkage method and the cosine of the angular distance. Jinshuibao capsules, Jinshuibao tablets, and Bailing capsules are referred to A, B, and C, respectively, for simplicity. The results are shown in Figure 5. The 30 samples were categorized into two groups: one containing 20 samples (Group 1) and the other containing 10 samples (Group 2). Group 1 was further divided into Subgroups 1a (A2, A3, A4, A10, B1, B2, B3, B4, B5, B6, and B10), 1b (A1, A5, A6, A7, A8, and A9), and 1c (B17, B18, and B19). Subgroup 1a contained 4 Jinshuibao capsules and 7 Jinshuibao tablet samples, Subgroup 1b contained 6 Jinshuibao capsules, and Subgroup 1c contained 3 Jinshuibao tablets. All Bailing capsule samples were grouped together. The 11 samples with cosine and correlation coefficient values between 0.989 and 0.998 are in Subgroup 1a, the 6 samples with values greater than 0.998 are in Subgroup 1b, and the 3 samples with values less than 0.986 are in Subgroup 1c. These groupings implied that HCA was effective for identifying *Cordyceps sinensis* samples fermented with different bacterial strains.

### 3.5. The Quantitative Analysis of Multicomponents by Single Marker (QAMS)

The RCF values of the four analytes were calculated at different injection volumes (2, 4, 6, 8, 10, 12, and 15 *µ*L) and are shown in Table 4. Many factors impacted the RCF values. In this work, the flow rate, column temperature, type of reversed-phase column, and chromatographic system were optimized to investigate the robustness of the RCFs, and the results are shown in Tables 5–7. The values were found to be accurate and stable.

### 3.6. Comparison of the Result of QAMS with Those of the External Standard Method (ESM)

To assess the differences in the results of the QAMS and ESM methods, 30 samples, including 10 Jinshuibao capsules, 10 Jinshuibao tablets, and 10 Bailing capsules, were analysed. Vector angles were used to determine the consistency of the results. The larger the cosine of the angle, the higher the similarity between the two samples. The results are shown in Table 8, and the values of the cosine of all the angles were more than 0.999, which indicated there were no significant differences between the results of QAMS and ESM. From these results, we can see that all samples contained all five active ingredients, but their contents are different in samples fermented with different strains; for example, for the Jinshuibao capsules (adenosine > uridine > guanosine > adenine > uracil), Jinshuibao tablets (adenosine > uridine > guanosine > adenine > uracil), and Bailing capsule (adenosine > guanosine > uridine > uracil > adenine). As a whole, the contents of adenosine, uridine, and guanosine were relatively high and met the standard given by the Pharmacopoeia of China.

## 4. Conclusion

The results presented herein suggest that fingerprint chromatography based on HPLC is an effective method for assessing the quality of fermented *Cordyceps sinensis* products, and the QAMS method is convenient and reliable for qualitifying the five active components in fermented *Cordyceps sinensis* by RCF. When HPLC fingerprint analysis was combined with the SA, HCA, and QAMS methods, it was a practical, feasible, and effective approach to better identifying and comprehensively evaluating the quality of fermented *Cordyceps sinensis* products. Hence, the method established herein is a promising and broadly applicable strategy for the comprehensive quality control of TCMs.

## Figures and Tables

**Table 1:** Gradient elution program.

*T* (min)	A (%)	B (%)
0	99	1
15	99	1
30	85	15

**Table 2:** Calibration curves for the five target compounds.

Peak number	Analyte	Calibration curve	*R* ^2^	Linear range (*µ*g/mL)	LOD (*µ*g/mL)	LOQ (*µ*g/mL)
1	Uracil	*Y* = 41.219*X* + 5.2065	0.9997	0.338–33.8	0.005	0.025
2	Uridine	*Y* = 23.568*X* − 1.2283	1.0000	0.936–93.6	0.016	0.049
3	Adenine	Y = 62.462*X* + 4.2509	0.9998	0.301–30.1	0.001	0.005
4	Guanosine	*Y* = 22.077*X* − 27.477	0.9992	1.208–120.8	0.038	0.151
5	Adenosine	*Y* = 28.112*X* + 2.0196	1.0000	0.936–93.6	0.024	0.073

**Table 3:** Precision, repeatability, stability, and recovery of makers in Jinshuibao capsules (*n*=6).

peak number	Analyte	Intraday RSD (%)	Interday RSD (%)	Repeatability RSD (%)	Stability RSD (%)	Recovery (%)
1	Uracil	0.16	0.10	0.55	0.19	97.5
2	Uridine	0.42	0.05	0.57	0.19	96.7
3	Adenine	1.10	0.78	1.36	0.48	102.1
4	Guanosine	1.61	0.31	0.61	0.13	95.9
5	Adenosine	0.14	0.13	0.23	0.22	96.0

**Table 4:** The values of RCF based on different injection volumes (*n*=3).

Injection volume	*f*_1_	*f*_2_	*f*_3_	*f*_4_
2	0.702	1.227	0.482	1.384
4	0.706	1.235	0.474	1.359
6	0.709	1.240	0.486	1.398
8	0.711	1.245	0.474	1.387
10	0.712	1.249	0.471	1.385
12	0.719	1.267	0.480	1.410
15	0.717	1.254	0.478	1.398
Mean	0.711	1.245	0.478	1.389
RSD (%)	0.83	1.05	1.09	1.15

*f*_1_: *f*_uracil/adenosine_; *f*_2_: *f*_uridine/adenosine_; *f*_3_: *f*_adenine/adenosine_; and *f*_4_: *f*_guanosine/adenosine_.

**Table 5:** The values of RCF based on different columns and instrument conditions (*n*=3).

Instrument	Column	*f*_1_	*f*_2_	*f*_3_	*f*_4_
Agilent 1100	Phenomenex	0.674	1.193	0.501	1.281
Agilent 1100	Agilent	0.676	1.189	0.461	1.300
Agilent 1260	Phenomenex	0.685	1.200	0.444	1.32
Agilent 1260	Agilent	0.712	1.249	0.471	1.385
Waters 2695	Phenomenex	0.676	1.203	0.432	1.269
Waters 2695	Agilent	0.693	1.199	0.451	1.306
Mean		0.686	1.206	0.460	1.310
RSD (%)		1.46	2.19	2.42	4.09

*f*_1_: *f*_uracil/adenosine_; *f*_2_: *f*_uridine/adenosine_; *f*_3_: *f*_adenine/adenosine_; and *f*_4_: *f*_guanosine/adenosine_.

**Table 6:** RCFs values based on different flow rates (*n*=3).

Flow rate	*f*_1_	*f*_2_	*f*_3_	*f*_4_
0.9 mL/min	0.674	1.189	0.443	1.300
1.0 mL/min	0.693	1.199	0.451	1.306
1.1 mL/min	0.676	1.193	0.443	1.306
Mean	0.681	0.194	0.446	1.304
RSD (%)	1.04	0.50	0.46	0.35

*f*_1_: *f*_uracil/adenosine_; *f*_2_: *f*_uridine/adenosine_; *f*_3_: *f*_adenine/adenosine_; and *f*_4_: *f*_guanosine/adenosine_.

**Table 7:** RCFs based on different column temperatures (*n*=3).

Column temperature	*f*_1_	*f*_2_	*f*_3_	*f*_4_
25°C	0.677	1.195	0.445	1.310
30°C	0.693	1.199	0.451	1.306
35°C	0.676	1.194	0.443	1.290
Mean	0.682	1.196	0.446	1.302
RSD (%)	0.95	0.26	0.42	1.06

*f*_1_: *f*_uracil/adenosine_; *f*_2_: *f*_uridine/adenosine_; *f*_3_: *f*_adenine/adenosine_; and *f*_4_: *f*_guanosine/adenosine_.

**Table 8:** Comparison of the results from the QAMS and ESM of fermented *Cordyceps sinensis* products (mg·g^−1^).

Sample number	Uracil		Uridine		Adenine		Guanosine		Adenosine
	QAMS	ESM	QAMS	ESM	QAMS	ESM	QAMS	ESM	ESM
1	0.1393	0.1341	2.504	2.398	0.3191	0.3016	2.593	2.404	2.670
2	0.2357	0.2269	2.540	2.432	0.3891	0.3677	2.565	2.379	2.784
3	0.2220	0.2138	2.810	2.694	0.3530	0.3336	2.695	2.466	2.851
4	0.2235	0.2151	2.368	2.267	0.2805	0.2652	2.287	2.121	2.476
5	0.1123	0.1081	1.899	1.189	0.2468	0.2332	1.950	1.808	2.275
6	0.1275	0.1228	2.535	2.427	0.2694	0.2417	2.653	2.460	2.982
7	0.1326	0.1276	2.517	2.409	0.2913	0.2753	2.464	2.284	2.740
8	0.1100	0.1058	2.582	2.518	0.2439	0.2305	2.620	2.430	3.009
9	0.1404	0.1351	1.997	1.192	0.2219	0.2097	2.180	2.021	2.518
10	0.2235	0.2151	3.024	2.896	0.3798	0.3590	2.574	2.387	2.714
11	0.1599	0.1539	1.573	1.506	0.2369	0.2264	1.547	1.434	1.583
12	0.1646	0.1585	1.624	1.555	0.2479	0.2343	1.591	1.475	1.629
13	0.0999	0.0961	1.775	1.670	0.1984	0.1875	1.650	1.530	1.745
14	0.1617	0.1557	1.593	1.525	0.2394	0.2262	1.558	1.444	1.595
15	0.1149	0.1106	2.039	1.925	0.2301	0.2174	1.861	1.726	1.957
16	0.1278	0.1231	1.718	1.645	0.2090	0.1975	1.674	1.552	1.752
17	0.1101	0.1065	2.335	2.236	0.2277	0.2152	1.698	1.575	1.858
18	0.1099	0.1058	2.295	2.197	0.2091	0.1977	1.665	1.544	1.828
19	0.1143	0.1100	2.385	2.284	0.2406	0.2273	1.737	1.611	1.896
20	0.1077	0.1031	1.850	1.772	0.2059	0.1946	1.762	1.634	1.830
21	0.1098	0.1057	1.158	1.109	0.0785	0.0742	2.241	2.078	2.269
22	0.1068	0.1028	1.283	1.229	0.0834	0.0789	2.325	2.156	2.207
23	0.1023	0.0985	1.067	1.022	0.0756	0.0714	1.946	1.804	1.684
24	0.0914	0.0879	0.737	0.707	0.0597	0.0564	1.524	1.413	1.370
25	0.0897	0.0864	0.974	0.932	0.0925	0.0875	2.111	1.958	1.901
26	0.1028	0.0989	1.249	1.197	0.0636	0.0599	2.251	2.088	2.257
27	0.1569	0.1510	1.697	1.625	0.1281	0.1208	3.048	2.827	2.755
28	0.1401	0.1348	1.532	1.467	0.1211	0.1145	2.689	2.493	2.538
29	0.0851	0.0819	0.969	0.928	0.0741	0.0701	1.893	1.756	1.816
30	0.1007	0.0969	1.245	1.192	0.0711	0.0672	2.526	2.343	2.595
Correlation coefficient	0.9999		0.9998		0.9997		0.9999		—
